# How well do elderly patients with major depressive disorder respond to antidepressants: a systematic review and single-group meta-analysis

**DOI:** 10.1186/s12888-020-02514-2

**Published:** 2020-03-04

**Authors:** Katharina Gutsmiedl, Marc Krause, Irene Bighelli, Johannes Schneider-Thoma, Stefan Leucht

**Affiliations:** Department of Psychiatry and Psychotherapy, School of Medicine, Klinikum rechts der Isar, Technical University of Munich, Munich, Germany

**Keywords:** Depression, Elderly, Response, Systematic review

## Abstract

**Background:**

Depression is one of the leading causes of the global burden of disease, and it has particularly negative consequences for elderly patients. Antidepressants are the most frequently used treatment. We present the first single-group meta-analysis examining: 1) the response rates of elderly patients to antidepressants, and 2) the determinants of antidepressants response in this population.

**Methods:**

We searched multiple databases for randomized controlled trials on antidepressants in the elderly with major depressive disorder above 65 years (last search: December 2017). Response was defined as 50% improvement on validated rating scales. We extracted response rates from studies and imputed the missing ones with a validated method. Data were pooled in a single-group meta-analysis. Additionally, several potential moderators of response to antidepressants were examined by subgroup and meta-regression analyses.

**Results:**

We included 44 studies with a total of 6373 participants receiving antidepressants. On average, 50.7% of the patients reached a reduction of at least 50% on the Hamilton Depression Scale (HAMD). Subgroup and meta-regression analyses revealed a better response to treatment for patients in antidepressant-controlled trials compared to placebo-controlled trials. Mean age, study duration, percentage of woman, severity of illness at baseline, dose of antidepressants in fluoxetine equivalents, year of publication, setting (in- or out-patients), antidepressant groups (SSRI, TCA, SSNRI, α2-antagonist, SNRI, MAO-inhibitor), ITT (intention-to-treat) analysis vs completer analysis, sponsorship and overall risk of bias were not significant moderators of response.

**Conclusions:**

Our findings suggest an improvement in symptoms can be found in about 50% of the elderly with major depressive disorder treated with antidepressants.

## Background

In high-income regions such as western Europe and North America, major depression is one of the most common causes for the burden of disease [1]. In the period from 1990 to 2013, major depression worldwide rose from 15th place to 11th place among the leading causes of burden of disease [1]. Indeed, the estimated lifetime prevalence of depression in the elderly over the age of 65 years is 9.6% for men and 20.4% for women [2]. Major depression can be associated with significant disability and reduced quality of life [3]. In addition, there is an increased suicide rate in patients suffering from major depression. Up to 15% commit suicide [4]. Especially in the elderly population rates of suicide are high and as the proportion of the elderly will increase in the future, the number of suicides will also rise [5].

Antidepressants are the most frequently used treatment for major depression [6].. A recent comprehensive meta-analysis showed efficacy in the 18 to 65-year-old population compared to placebo [6]. However, antidepressant’s effect on the subpopulation of the elderly is still unclear. Specific evidence for this subgroup is highly required, because elderly patients differ substantially from the adult age group, especially in terms of the efficacy and safety of pharmacological interventions, what necessarily should lead into an individualized treatment. These differences include age-related physiological changes which affect the pharmacodynamics, pharmacokinetics [7], high multimorbidity, an elevated probability of drug interactions due to polypharmacy [8] and differences in life circumstances.

Due to differences of pharmacokinetics, the effect on antidepressants might be peculiar in this population. Several metabolic changes in the body of old people could influence the concentration of active substance in the body by altering metabolism and elimination. For example, liver mass and blood flow decline and the creatinine clearance of the kidney decreases continuously with increasing age [9]. In addition, frequent polypharmacy in elderly patients increases the risk of drug interactions [8]. Therefore, concentrations of antidepressants should be chosen carefully to avoid overdosing, which could cause severe side effects. The pharmacodynamics could also change with age, as an increased pharmacodynamic sensitivity in the elderly, due to neuronal and neurotransmission changes [10, 11] was reported. Especially white matter lesions could play an important role, as they increase with age [11]. White matter hyperintensities on magnetic resonance imaging are found to be associated with a higher probability of chronicity of depression and a poorer response to antidepressant therapy [12]. Another aspect is the high level of comorbidity in older people. The response to antidepressants deteriorates in the presence of chronic illnesses that lead to reduced function or disability [13]. Moreover, the social situation of elderly patients can differ from younger adults in many factors that may hinder or facilitate to cope with depressive episodes and thus influence absolute response rates.

For this reason, our group conducted a comprehensive systematic review and network meta-analysis to compare different pharmacological and non-pharmacological treatments with each other in the elderly with major depressive disorder (MDD) [14].

In addition to estimates of the relative efficacy between different treatments, estimates of absolute efficacy (e.g. response rates) are needed also to understand the clinical relevance of relative differences between treatments, and to inform patients and clinicians about the expected average outcome with a given treatment. Results in the form of response rates are relevant especially for clinical practice, because they represent a pragmatic outcome that can be interpreted easily by practitioners.

However, numbers of patients improving with the treatment are not always reported in the studies. Also, there is currently no comprehensive evidence synthesis which focuses on this question in geriatric patients.

To fill this gap, we addressed this point applying a validated methodology [15–17] to calculate response rates from rating scales’ measures. The estimated response rates of all antidepressant-study-arms from different studies were combined meta-analytically to provide an estimate of the average response rate on antidepressants. This methodology distinguishes this work from most meta-analyses, which focus typically on the relationships between the interventions and comparison groups, such as the network meta-analysis conducted previously by our group [14].

Therefore, we present the first systematic review and single-group meta-analysis of response rates in elderly patients with a major depressive disorder who participated in randomized controlled trials. The purpose of this work is twofold: 1) to calculate how well elderly patients with a major depressive disorder respond to antidepressants; 2) to investigate the determinants of antidepressant response in this population.

## Methods

The present meta-analysis is part of a comprehensive project to treat elderly, depressed people. The project’s protocol has been published in PROSPERO (CRD 42018107814). The project includes a network meta-analysis [14] that examined all types of antidepressants for geriatric patients. The methods for search strategies, inclusion criteria, data extraction, and the evaluation of the risk of bias described in the protocol also apply to the current work.

### Search strategy and study inclusion criteria

**Fig. 1:**
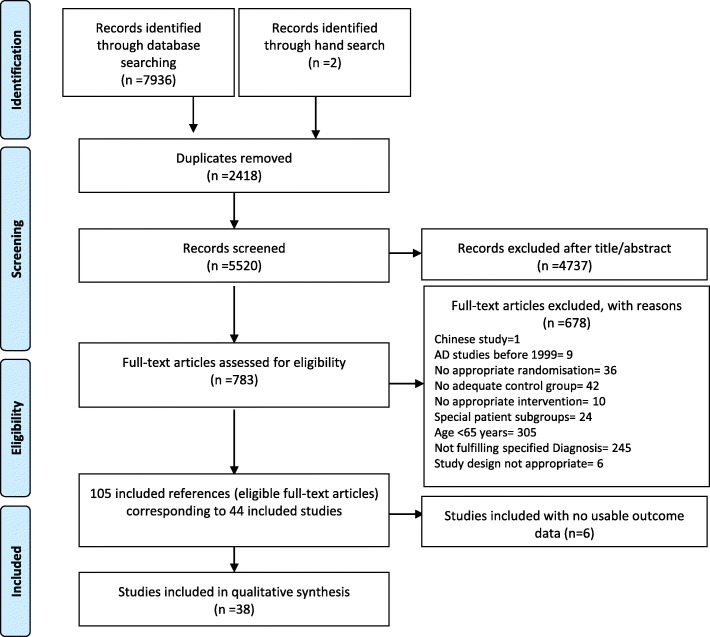
Study selection process PRISMA flow-chart

We identified randomised controlled trials (RCTs) in elderly with acute major depressive disorder through a comprehensive, systematic literature search in the specialised register of the Cochrane common mental disorders group, MEDLINE, EMBASE, PsycINFO, Cochrane Library, ClinicalTrials.gov and the WHO clinical trials platform until Dec 12, 2017. Moreover, we inspected the reference lists of the included studies and previous reviews [18–21]. We only included studies that applied an operationalised diagnosis of Major Depressive Disorder (MDD) and excluded studies where all patients had a specific comorbidity per inclusion criteria. For example, studies were excluded, if they only dealt with depression in patients with diabetes. However, patients in the included studies still could have general physical comorbidities, making the included population representative for the elderly group. We defined the subgroup of elderly patients according to the definition of the “German Society of Geriatrics” (DGG) [22] with a minimum age of 65 (leads to a mean age of over 70) to make sure that the patient characteristics differ from general adult patients. We excluded studies published before 1990 to take into account the change in placebo response from this time identified in studies about depression [23]. In studies with a crossover design only the first crossover phase was used in order to avoid the problem of carryover effects [24]. Cluster-randomized studies and studies with a high risk of bias for sequence generation were excluded [25, 26]. Double-blind studies not explicitly mentioning randomization were assumed to be randomized. Two reviewers (MK, KG) independently assessed the Study quality using the Cochrane Collaboration’s risk-of-bias tool [26]. Studies from mainland China were excluded, as many of these studies have been reported to be not reliable, do not use appropriate randomization procedures and do not report their methods [27–32]. Only one study was excluded for this reason (see Fig. 1). For missing data, we sent emails to the first or corresponding authors of the included studies.

### Screening and data extraction

Study selection and data extraction were performed independently by at least 2 reviewers (KG, MK). Disagreement was resolved by discussion or, in case of need, by involving the team leader (SL) or contacting the authors for additional information. Risk of bias was assessed independently also by at least 2 reviewers (MK, KG) using the Cochrane risk-of-bias tool which contains the items’ sequence generation, allocation concealment, blinding, completeness of outcome data, selective reporting and other bias.

### Definition of response

In depression trials response is usually defined as a 50% reduction on the Hamilton Depression Scale (HAMD) [33] or Montgomery-Asberg-Depression Scale (MADRS) [34] total score from baseline to endpoint. According to equipercentile-linking studies that compared MADRS/HAMD ratings with simultaneous Clinical Global Impression (CGI) ratings, a score of at least 50% reduction from baseline approximately means ‘much improved’ measured with the CGI [35, 36].

In studies were response rates were not reported, an imputation method proposed by Furukawa and colleagues [17] that has already been applied successfully [16, 15] was used to estimate at least 50% reduction from baseline based on means and standard deviations at endpoint of the HAMD or their change scores from baseline.

### Data analysis

As already mentioned, this work differs from most meta-analyses as we conducted a single-group summary meta-analysis. The focus was not on the relationship between the interventions, but on the response rate in a particular population. For this purpose, the response rates of the individual study arms were pooled, without differentiating the various drugs. As a result, the average response rate in elderly with MDD treated with antidepressants could be provided. We used the Comprehensive Meta-Analysis software (version 2.0) (Biostat, Inc.,Englewood, NJ.USA) for the calculations. The meta-analytical calculations for obtaining an average of all studies are essentially the same, regardless of whether it is a single-group summary or a between-group difference [37]. The intention-to-treat datasets was used for the analyses. We also conducted a sensitivity analysis to detect possible changes in the results when excluding studies with imputed response rates [17].

For assessing heterogeneity the I-square statistic was used as proposed by Higgins and Colleagues [38], with values > 50% indicating a considerable heterogeneity. Subgroup analyses for dichotomous outcomes were conducted using a mixed-effect model and meta-regression analyses for continuous outcomes were conducted using a random-effect model. The aim of these analyses was to identify study characteristics that contributed to the heterogeneity. For the meta-regression analyses the following moderators were chosen a priori: percentage female participants, mean age, baseline severity, mean daily dose in fluoxetine equivalents [39], study duration and year of publication. To assess baseline severity, the different depression scales had to be made comparable. To achieve that we converted the MADRS scores into HAMD scores using the conversion table from Leucht et al. [40]. As there are HAMD scales with a different number of items used in the RCTs we divided them by their stated item number and multiplied them by 17 to estimate scores based on an HAMD-17 item scale.

Subgroup analyses were performed by grouping studies according to the antidepressant subgroups (SSRI, TCA, SSNRI, SNRI, α2-Antagonit, MAO-Inhibitor), the setting (in- or out-patients), the sponsorship, the presence of a placebo arm, ITT analysis vs completer analysis, overall risk of bias and blinding (double-blind or open studies). The division into the antidepressant groups was carried out with the help of neuroscience-based nomenclature [41] and the Duale Reihe Pharmakologie und Toxikologie [42]. The difference between ITT and completer analysis is that ITT analysis includes all randomized patients from the beginning of a study [43] whereas completer analyses only include participants who remained until the end of the study. Studies received an overall high risk-of-bias status if two or more out of the six items, proposed by the Cochrane risk-of-bias tool, were judged as high risk [44].

As there was only a small number of studies available for each included drug, the effect of single drugs could not be meaningfully assessed.

The issue of small study effects was considered by visually examining the funnel plot and by conducting the Egger’s test for funnel-plot asymmetry.

## Results

### Description of included studies

We identified 7938 citations through the literature search and 5520 references were left after duplicates were removed. After excluding irrelevant reports by reviewing the titles and abstracts, 783 potentially eligible articles were retrieved in full text. Forty-four studies with a total of 6373 participants receiving antidepressants were included in the analysis. A PRISMA flowchart is presented in Fig. 1. Description of included studies is presented in Additional file 1.

Of the 44 included studies, 33 studies reported a response rate. In 5 studies response rates were imputed. The median study duration was 9 weeks (range 4–12). The mean age of participants was 73.9 years (s.d. = 2.96). The mean baseline severity (HAMD equivalent) was 22.58. The antidepressant involved in most comparisons was paroxetine (8 of 44 trials) followed by fluoxetine (7 of 44 trials) whereas few trials were available for most other antidepressants: mianserin (5), amitriptylin (4), citalopram (4), duloxetine (4), sertraline (4), venlafaxine (4), dothiepin (3), fluvoxamine (3), imipramine (3), escitalopram (2), reboxetine (2), tianeptine (2), and only one single study for the drugs: agomelatine, bupropion, clomipramine, doxepin, lofepramine, maprotiline, milnacipran, mirtazapine, moclobemide, nortriptylin, trazodone, trimipramine, vortioxetine.

The mean dosage of antidepressants in fluoxetine equivalents [39] was 30.21 mg/day. Figures illustrating the risk-of-bias assessment are presented in the Additional file 2. The trial reports often did not provide details about randomisation procedures (80%) nor allocation concealment (80%). The blinding of patients and personnel was unclear in 45% of the studies and showed a high risk in 5%. The risk of bias for blinding of outcome assessment was similar with 45% unclear and 5% high risk. The rates of high risk of bias for missing outcomes and selective reporting were 5 and 9% respectively and an unclear risk for these variables was found in 9 and 11% respectively. There were no serious other biases which could affect our results.

### Response rates

**Fig. 2:**
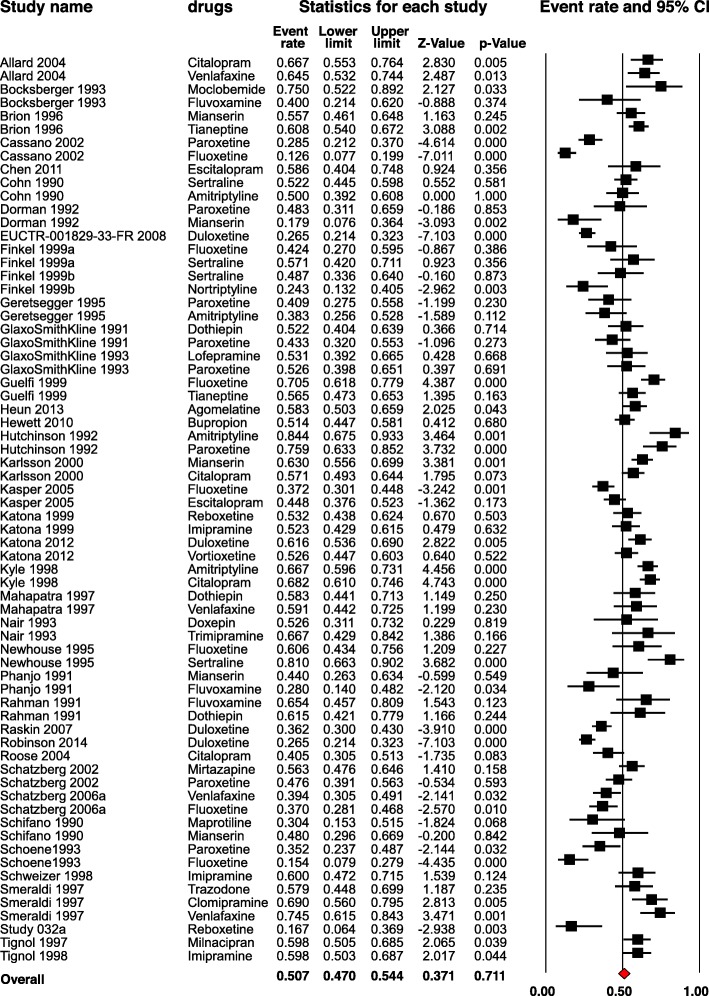
Pooled results for response rate of 50% reduction from baseline, random-effects model. The squares represent the response rates and the horizontal lines reflect the 95% confidence interval. The red diamond corresponds to the overall response rate. Cl = Confidence interval

The pooled response rate for the cut-off, at least 50% HAMD reduction from baseline, was 50.7% (38 RCTs, 5991 participants, 95% CI 47.0 to 54.4%, I^2^ = 86.2%) (Fig. 2). The analysis revealed considerable heterogeneity in the response rates between the different studies, and we conducted subgroup and meta-regression analyses in order to find possible explanations.

### Sensitivity analysis

In the sensitivity analysis 5 studies [45–49] with imputed response data were excluded. The average response rate for 50% reduction of HAMD in elderly patients with major depressive disorder was 51.7% (see Additional file 3).

### Meta-regression analyses (Table 1)

#### Percentage female participants

The meta-regression with percentage of female as a moderator suggested that female patients might have a better clinical response than males (slope = 0.01). However, the result was just not statistically significant (*p* = 0.06).

**Table 1:** Meta-regression (continuous moderators)

*Moderator*	*Coefficient*	*Lower limit*	*Upper limit*	*Z-value*	*P-value*
Percentage female participants (*N* = 57)	0.01	−0.00	0.03	1.90	0.06
Baseline severity (*N* = 60)	0.02	−0.03	0.07	0.89	0.37
Mean age (*N* = 66)	−0.05	−0.11	0.02	−1.43	0.15
Mean daily dose in fluoxetine equivalents (*N* = 31)	−0.00	−0.02	0.02	−0.37	0.71
Study duration (*N* = 68)	0.01	−0.06	0.08	0.32	0.75
Publication Year (N = 68)	−0.01	−0.04	0.01	−0.98	0.33

N: Number of study arms.

#### Baseline severity

The meta-regression did not suggest a role of baseline severity in moderating response rates (*p* = 0.37).

#### Mean age

Response rate was not found to be associated with mean age of participants (slope = − 0.05, *p* = 0.15).

#### Mean daily dose in fluoxetine equivalents

Response rate was not found to be associated with dosage in fluoxetine equivalents (*p* = 0.71).

#### Study duration

Response rate was not found to be associated with study duration (*p* = 0.75).

#### Year of publication

Response rate was not found to be associated with publication year (*p* = 0.33).

### Subgroup analyses (Table 2)

#### Antidepressant subgroups (Fig. 3)

The tests for subgroup differences of response rates between SSRI, SSNRI, TCA, SNRI, MAO-Inhibitor and α2-Antagonist were not statistically significant, except for the two comparisons MAO-Inhibitors with SSRI (*p* = 0.027) as well as with SSNRI (*p* = 0.047) (Response Rates: SSRI = 48.1%, SSNRI = 49.0%, TCA = 56.5%, SNRI = 34.0%, α2-Antagonist = 50.6%, MAO-Inhibitor = 75.0%). The comparison of SSRI and TCA was just not statistically significant (*p* = 0.06). For the remaining *p*-values ​​for the tests on subgroups difference see Additional file 4.

**Table 2:** Subgroup analyses (dichotomous moderators)

*Moderator*	*Percentage responders*	*Lower limit*	*Upper limit*	*Q-value for subgroup differences*	*P-value for subgroup differences*
Antidepressant subgroups					
SSRI (*N* = 28)	0.48	0.42	0.54		
TCA (*N* = 15)	0.57	0.50	0.63		
SSNRI (*N* = 9)	0.49	0.37	0.61		
α2-Antagonist (N = 6)	0.51	0.41	0.60		
SNRI (N = 2)	0.34	0.09	0.74		
MAO-Inhibitor (N = 1)	0.75	0.52	0.89		
Setting In/ Outpatients					
Inpatients (N = 9)	0.46	0.35	0.57	0.39	0.53
Outpatients (*N* = 37)	0.50	0.44	0.55		
Sponsorship					
Sponsor stated (*N* = 39)	0.51	0.46	0.57	0.10	0.75
No sponsor stated (*N* = 29)	0.50	0.45	0.55		
Presence of a placebo arm					
Placebo comparison (N = 15)	0.43	0.37	0.50	6.35	0.01
No Placebo comparison (*N* = 53)	0.53	0.49	0.57		
Overall high risk of bias					
Studies without overall high risk of bias (*N* = 65)	0.51	0.47	0.55	0.19	0.67
Studies with overall high risk of bias (N = 3)	0.44	0.18	0.74		
Data analysis					
ITT (N = 53)	0.53	0.49	0.57	1.22	0.27
Completer analyses (N = 5)	0.40	0.21	0.63		

N = Number of study arms.

**Fig. 3:**
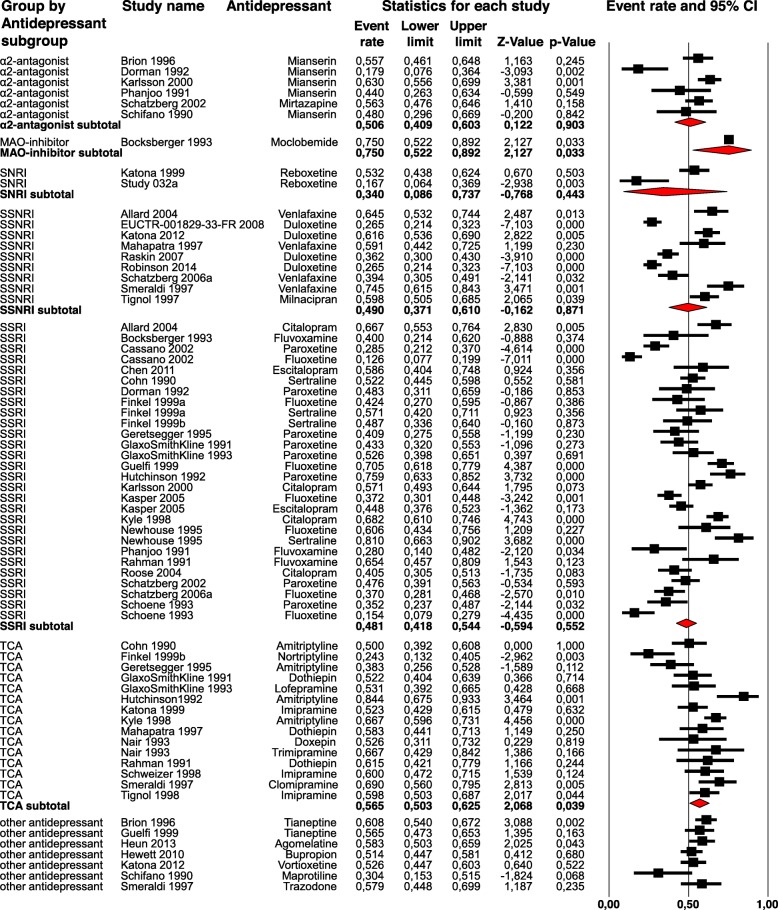
Forest Plot - Response rates grouped by antidepressant subgroup. The squares represent the response rates and the horizontal lines reflect the 95% confidence interval. The red diamond corresponds to the subtotal response rate in the different antidepressant groups. MAO-inhibitors = Monoamine oxidase inhibitors, SNRI = Serotonin–norepinephrine reuptake inhibitor, SSNRI = Selective serotonin–norepinephrine reuptake inhibitor, SSRI = Selective serotonin reuptake inhibitor, TCA = Tricyclic antidepressant, CI = Confidence interval

#### In/outpatients

The test for subgroup differences of response rate between in- and outpatients was not statistically significant (46% vs 50%, *p* = 0.53).

#### Sponsorship

The test for subgroup differences of response rate between studies with a pharmaceutical sponsor manufacturing a drug and studies with no such sponsor stated was not statistically significant (51% vs 50%, *p* = 0.75).

#### Presence of a placebo arm

We found a statistically significant lower response rate in studies with a placebo arm compared to studies with exclusive active treatments (43% vs 53%, *p* = 0.01).

#### ITT analysis vs completer analysis

The test for subgroup differences of response rate between studies with ITT analysis and studies with completer analysis was not statistically significant (53% vs 40%, *p* = 0.27).

#### Overall risk of bias

The test for subgroup differences of response rate between studies with an overall high risk of bias and studies without an overall high risk of bias was not statistically significant (44% vs 51%, *p* = 0.67).

#### RCTs blinding status

There was only one open RCT, as this study did not provide any usable data, no analysis could be performed.

### Small-study effect

There was no obvious asymmetry in the funnel plot, which would have indicated small-study effects. This was also confirmed by a formal test of funnel plot asymmetry see Additional file 5 [50].

## Discussion

To the best of our knowledge, this is the first systematic review and meta-analysis that evaluates how well elderly patients with major depressive disorder respond to antidepressants based on all available randomized controlled trials.

In summary, our results show that 50.7% of elderly patients with major depressive disorder responded while treated with antidepressants. Subgroup and meta-regression analyses revealed a better response to treatment for patients in antidepressant-controlled trials compared to placebo-controlled trials. Mean age, study duration, percentage of woman, severity of illness at baseline, dose of antidepressants in fluoxetine equivalents, year of publication, setting (in- or out-patients), antidepressant groups, ITT analysis vs completer analysis, sponsorship and overall risk of bias were not significant moderators of response.

Our main finding was that 50.7% of elderly patients with major depressive disorder reached a reduction of depressive symptoms of at least 50% from baseline. Compared to a recent meta-analysis by Kok et al. [18] the response rates were similar (48% vs. 51%), even though Kok et al. used very broad inclusion criteria, with a lower age limit of 55 years, and thus potentially including younger patients, which do not show typical geriatric characteristics. We focused on patients classified as geriatric by recent definitions, e.g. of the “German Society of Geriatrics” (DGG), or the “German Society of Gerontology and Geriatrics” (DGGG) [22], including mainly an age over 70 and a high multimorbidity. However, since there was no great difference between the response rates of the two meta-analyses, the chronological age may play a smaller role. A recent study showed that the association of biological age with depression is superior to chronological age [51].

Another recent meta-analysis by Tedeschini et al. included a subgroup analysis for patients over 65, based only on six studies and only included placebo-controlled trails [21]. Tedeschini and colleagues found a considerably lower response rate (42.4%) for this age group. They also reported response rates for late life MDD with a lower age limit of 55 years (45.2%) [21]. Despite the different age limits, the response rates are similar, which emphasizes the hypothesis that the chronological age is not decisive. The reason for the overall lower response rate in Tedeschini et al. is probably caused by the fact that they only included placebo-controlled studies in the analysis. As already reported in the results, we found a statistically significant lower response rate in studies with a placebo arm compared to studies with exclusive active treatments. Our analysis showed a response rate similar to Tedeschini et al. of 43.1% (vs 42.4%) for placebo-controlled studies.

Interestingly, we found no meaningful difference between the response rates of the elderly population and adults between the ages of 18 and 65 years. Based on the published dataset by Cipriani and colleagues [6] we calculated a response rate of 53.0% for the adult population (vs 50.7% in the elderly) (see Additional file 6). There are arguments in the literature that indicate a poorer response rate among older people. Studies have shown that structural changes in the brain, such as white-matter hyperintensity, lead to a poorer response rate to antidepressants, and since white-matter hyperintensity occurs primarily in old age, it should particularly affect this age group [52, 53]. Also, the higher comorbidity in the elderly should play an important role. Studies found that physical illness and depression have a negative impact on each other [13, 54]. On the contrary, as already mentioned in the introduction section, metabolic changes in the elderly could possibly lead to higher doses of active substance in the organism [9]. This could lead to a higher risk of side effects, but also to a higher response to the medication. Moreover, differences in social circumstances (having an impact on the disease and the treatment) between elderly and younger adults need to be taken into account. We conclude that either differences in age do not have a strong impact on response to antidepressant treatment, or that the factors that could lead to a higher or to a lower response in the elderly population in the end compensate each other.

Although the aim of this study was not to compare antidepressant response rates to placebo response rates, we calculated, as additional information, the response rate for patients in the placebo arm, which we found to be equal to 31.7% (see Additional file 7). This result is in line with Cipriani and colleagues finding in their recent, comprehensive meta-analysis that all analysed antidepressants were more efficacious than placebo in adults with major depressive disorder [6].

The almost significant result in relation to gender is in line with the findings of previous work [55, 56], which found increased treatment response in female patients, especially the ones treated with SSRIs. Since all included drugs were used for this meta-regression and no further subdivision in antidepressant groups was made, no statement can be made as to whether women respond better to SSRIs than men.

Contrary to previous work of Calati et al., we did not find a role for severity of illness at baseline in moderating response rates [55].

Similarly, the meta-regression with mean age as a moderator did not show differences in response rates related to age. As we included patients with a minimum age of 65 years, the mean age ranges from 68.9 to 83.2 years, what is probably too small a range to show an effect in meta-regression analysis. In addition, biological age may play a greater role than chronological age in terms of antidepressant response [51]. However, since we could not find any difference in the response rates between adults and older people, see above, it is possible that age is not a decisive factor for the response rate.

There was no relationship between drug dosage and response rates. Doses of individual drugs had to be converted to fluoxetine equivalents for this purpose. All methods of dose equivalents have serious limitations [39].

Both moderators’ study duration and publication year did not reveal an association with response rates in the meta-regression analysis.

We found a significantly higher response rate in patients treated with MAO-Inhibitors compared to patients treated with SSRIs or SSNRIs. However, this finding has limited meaning as only one study could be included in the MAO-Inhibitor subgroup. The number of studies was also low in the subgroups SNRI (2 studies), α2 antagonists (6 studies) and SSNRI (9 studies).

We found that studies with antidepressant control groups had a significantly higher response rate than placebo-controlled trials. In the present analysis 31.6% of the 38 included studies were placebo-controlled trials. The knowledge of a possible placebo treatment could have affected the patients negatively. A similar effect was observed in a meta-analysis of Cipriani et al. [6]. The same drug achieved a higher efficacy in antidepressant-controlled trials, compared to placebo-controlled trials [6]. Furthermore, early withdrawal from the study was higher in placebo-controlled groups, which was associated with a lower response rate [6]. Another meta-analysis [57] also confirmed higher response rates in comparator trials compared to placebo-controlled trials. This finding has important implications for the execution and interpretation of placebo-controlled trials.

Finally, there were no significant differences of response rates in the subgroup comparisons of in−/out-patients, sponsorship, ITT analysis vs completer analysis, and overall risk of bias. The subgroups ITT vs completer and overall risk of bias were very different in the number of studies included (53 vs 5 and 65 vs 3), so the meaningfulness of both analyses is limited.

Several limitations should be considered while interpreting our results. Heterogeneity was high as our inclusion criteria allowed treatment with all currently available antidepressants and there was no restriction in terms of dosage. Furthermore, the included studies used different depression scales to define response criteria. In addition to the two most common scales: HAMD (20 studies) and MADRS (7 studies), response was reported also using the Geriatric Depression Scale (GDS) [58] (2 studies) and the Clinical Global Impression CGI [59] (4 studies). Apart from the CGI scale, response was defined as 50% reduction of baseline. Recent validation studies have shown that “much improved” on the CGI scale corresponds to a 50% reduction of the HAM-D/MADRS [35, 36], therefore no relevant differences in outcome were expected. Finally, further interesting moderators like duration of illness could not be analysed, as data was only reported in 4 of the 44 studies. A major strength of the analysis is the high number of participants (*n* = 5991 with usable outcome data), as it provides robust results. Other strengths of our analysis are the strict inclusion criteria. We only included randomised controlled trials with a minimum age of 65 years and whose patients had an operationalised diagnosis of MDD. We opted for the age limit of 65 years because this age group shows typically geriatric characteristics and a high multimorbidity [22]. To the quality of our analysis also contributes that only 5 studies did not report response rates and, therefore, required an imputation of response rates. The results from our sensitivity analysis excluding the imputed response (51.7%) were consistent with the main analysis (50.7%).

## Conclusions

Based on these results, we conclude that clinicians can expect that about 50.7% of elderly patients with major depressive disorder treated with antidepressants experience an improvement in symptoms. This response rate is not substantially different from the one in the general adult population.

## Supplementary information

**Additional file 1.** Included studies (pdf).

**Additional file 2.** Risk of bias assessment (pdf).

**Additional file 3.** Sensitivity analysis (pdf).

**Additional file 4 **Antidepressant subgroup *p*-values(pdf).

**Additional file 5.** Small-study effect (pdf).

**Additional file 6.** Response rates in adults with MDD (pdf).

**Additional file 7.** Placebo response rates (pdf).

## Data Availability

The datasets used and/or analysed during the current study are available from the corresponding author on reasonable request. More information about this project is available at the project website: https://www.psykl.mri.tum.de/node/73

## Abbreviations

CGI: Clinical Global Impression
GDS: Geriatric depression scale, HAMD: Hamilton Depression Scale
ITT: Intention to treat
MADRS: Montgomery–Åsberg Depression Rating Scale
MAO-inhibitor: Monoamine oxidase inhibitor
MDD: Major depressive disorder
RCT: Randomised controlled trial
SNRI: Serotonin norepinephrine reuptake inhibitor
SSNRI: Selective serotonin norepinephrine reuptake inhibitor
SSRI: Selective serotonin reuptake inhibitor
TCA: Tricyclic antidepressant
