# Supplementary material for: How well do elderly patients with major depressive disorder respond to antidepressants: a systematic review and single-group meta-analysis
Source: BMC Psychiatry. 2020 Mar 4;20:102. doi: 10.1186/s12888-020-02514-2 (PMC7057600; doi:10.1186/s12888-020-02514-2)
Supplement: Supplementary file 1 — Additional file 1. Included studies (pdf). [file 12888_2020_2514_MOESM1_ESM.pdf]

Characteristics of included studies

Study	Study groups/number of participants	Mean doses (Range) (mg/days)	Trial duration (weeks)	Mini mum Age	Mean Age	Diagnosis	Study design	Sponsors
Allard2004 (1)	Venlafaxine: n= 76<br>Citalopram: n= 75	V: 116 (75-150)<br>C: 26 (20-30)	8	64	73,1	DSM-IV major depression	DB-RCT	Wyeth Elderly
Anon 2003 (2)	Nortriptyline: n= 34<br>Venlafaxine: n= 34	N: 62,5 (50-100)<br>V: 251,47 (225-300)	26	65	70,83	DSM-IV unipolar major depression	SB-RCT	No sponsor stated
Bocksberger 1993 (3)	Fluvoxamine: n= 20<br>Moclobemide: n= 20	F: 172 (100-200)<br>M: 433 (300-450)	4	65	74,45	DSM-III major depressive episode	DB-RCT	No sponsor stated
Brion 1996 (4)	Tianeptine: n= 209<br>Mianserin: n= 106	T: 31,25 (25-37,5)<br>M: 30	26	70	78,53	DSM-III-R depression majeure	DB-RCT	No sponsor stated
Cassano 2002 (5)	Fluoxetine: n= 119<br>Paroxetine: n= 123	F: 20-66<br>P: 20-40	6	65	75,24	ICD-10 depression (paragraphs F32, F32.1 and F322	DB-RCT	SmithKline Beecham
Chen 2011 (6)	Escitalopram: n= 29<br>Placebo n= 26	E: 10	8	65	68,9	DSM-IV major depression	DB-RCT	No sponsor stated
Cohn 1990 (7)	Sertraline: n= 161<br>Amitriptyline: n= 80	S: 116 (50-200)<br>A: 88 (50-150)	8	63	70,33	DSM-III major depression or bipolar disorder	DB-RCT	No sponsor stated
Dorman 1992 (8)	Mianserin: n= 28<br>Paroxetine: n= 29	M: 60<br>P: 30	6	65	-	DSM-III unipolar depression	DB-RCT	No sponsor stated
EUCTR-001829-33-FR 2008 (9)	Placebo: n= 121<br>Duloxetine: n= 249	D: 60	12	65	72,89	DSM-IV-TR Major Depressive Disorder	DB-RCT	Eli Lilly
EUCTR-003821-25-DK 2005 (10)	Placebo: n= -<br>Escitalopram: n= 99	E: 10	12	65	70,3	ICD-10 depressive single episode, depressive recurrent episode or organic depressive episode	DB-RCT	No sponsor stated
EUCTR-005612-26-SK 2013 (11)	Escitalopram: n= 99<br>Placebo: n= 107<br>Tianeptine: n= 105	E: 10<br>T: 25-50	8	65	70,44	DSM-IV-T Major Depressive Episode	DB-RCT	Institut de Recherches Internationales Servier
Finkel 1999a (12)	Sertraline: n= 39<br>Nortriptyline: n=37	S: 102 (50-150)<br>N: - (25-100)	12	70	74,49	DSM-III-R major depression	DB-RCT	Pfizer

Finkel 1999b (13)	Fluoxetine: n= 33<br>Sertraline: n=42	F: 28,5 (20-40)<br>S: 72,6 (50-100)	12	70	74,44	DSM-III-R major depression	DB-RCT	No sponsor stated
Geretsegger 1995 (14)	Paroxetine: n= 44<br>Amitriptyline: n= 47	P: 20-30<br>A: 100-150	6	65	71,15	DSM-III major depression	DB-RCT	SmithKline Beecham
GlaxoSmithKline 1991a (15)	Paroxetine: n=6<br>Clomipramine: n= 5	P: 20-40<br>C: 25-100	14	65	83,2	DSM-III major depressive episode	DB-RCT	Glaxo-Smith-Kline
GlaxoSmithKline 1991b (16)	Dothiepin: n= 67<br>Paroxetine: n= 67	D: 75<br>P: 20	6	65	75,85	DSM-III-R major depressive episode	DB-RCT	Glaxo-Smith-Kline
GlaxoSmithKline 1993 (17)	Paroxetine: n=57<br>Lofepramine: n= 49	P: 20-30<br>L: 70-320	8	65	75,27	DSM-III-R major depressive episode	DB-RCT	Glaxo-Smith-Kline
Guelfi 1999 (18)	Tianeptine: n= 115<br>Fluoxetine: n=122	T: 25-37,5<br>F: 20	12	65	77,56	DSM-III-R major depressive episode	DB-RCT	Eli-Lilly
Heun2013 (19)	Placebo: n= 71<br>Agomelatine: n=151	A: 25 50	8	65	71,84	DSM-IV-TR moderate to severe episode of recurrent MDD	DB-RCT	Servier
Hewett 2010 (20)	Placebo: n= 208<br>Bupropion: n= 212	B: 179 (150-300)	10	65	71,10	DSM-IV MDD	DB-RCT	Glaxo-Smith-Kline
Hutchinson 1992 (21)	Amitriptyline: n= 32<br>Paroxetine: n= 58	A: 100<br>P: 30	6	65	71,82	DSM-III major depressive disorder	DB-RCT	Glaxo-Smith-Kline
Jansen 2003 (22)	Nortriptyline/ paroxetine	N: 25-72<br>P: 10-20	4	71	82	DSM-IV with major depressive or dysthymic disorders	DB-RCT	No sponsor stated
Karlsson 2000 (23)	Citalopram: n= 163<br>Mianserin: n= 173	C: 28 (20-40)<br>M: 40 (30-60)	12	64	75,17	DSM-III-R major depression	DB-RCT	No sponsor stated
Kasper2005 (24)	Fluoxetine: n= 164<br>Placebo: n= 180<br>Escitalopram: n= 174	F: 20<br>E: 10	8	65	75	DSM-IV MDD	DB-RCT	No sponsor stated
Katona 1999 (25)	Imipramine: n= 109<br>Reboxetine: n= 109	I: 50-100<br>R: 4-6	8	65	74,15	DSM-III-R MDD	DB-RCT	Pharmacia & Upjohn
Katona 2012 (26)	Duloxetine: n= 151<br>Vortioxetine: n= 156<br>Placebo: n= 145	D: 60<br>V: 5	8	65	70,57	DSM-IV MDD	DB-RCT	H. Lundbeck A/S
Kyle 1998 (27)	Citalopram: n= 179<br>Amitriptyline: n= 186	C: 24 (20-40)<br>A: 57 (50-100)	8	65	73,76	DSM-III-R major depression	DB-RCT	H. Lundbeck A/S
Mahapatra 1997 (28)	Dothiepin: n= 48<br>Venlafaxine: n= 44	D: 50-150<br>V: 50-150	6	64	74	DSM-III-R major depression	DB-RCT	wyeth ayerst pharmaceuticals

Nair 1993 (29)	Doxepin: n= 19<br>Trimipramine: n= 18	D: 138 (100-200)<br>T: 144 (100-200)	5	-	69,38	DSM-III Major Depressive Episode	DB-RCT	Rhone-Poulenc Rorer
NCT0013045 5 2006 (30)	Escitalopram/ Placebo	E: 5-10	12	65	-	ICD-10 single/recurrent / organic depressive episode	DB-RCT	Psychiatr ic Hospital, Hillerod
Newhouse 1995 (31)	Fluoxetine: n= 33<br>Sertraline: n= 42	F: 20-40<br>S: 50-100	12	70	74,4	DSM-III-R major depressive disorder	DB-RCT	Pfizer
Phanjoo 1991 (32)	Mianserin: n= 25<br>Fluvoxamine: n= 25	M: 60 (40-80)<br>F: 170 (100-200)	6	66	76,5	DSM-III major depressive episode	DB-RCT	No sponsor stated
Rahman 1991 (33)	Fluvoxamine: n= 26<br>Dothiepin: n= 26	F: 157 (100-200)<br>D: 159 (100-200)	6	61	74	DSM-III major depressive episode	DB-RCT	No sponsor stated
Raskin 2007 (34)	Placebo: n= 104<br>Duloxetine: n= 207	D: 60	8	65	72,86	DSM-IV recurrent major depressive disorder	DB-RCT	Eli Lilly
Robinson 2014 (35)	Duloxetine: n= 249<br>Placebo: n= 121	D: 60	12	65	73,04	DSM-IV-TR Major Depressive Disorder	DB-RCT	Eli Lilly
Roose 2004 (36)	Citalopram: n= 84<br>Placebo: n= 91	C: 10-40	8	75	79,59	DSM-IV unipolar depression, single or recurrent, nonpsychotic	DB-RCT	Forest
Schatzberg 2002 (37)	Mirtazapine: n= 128<br>Paroxetine: n= 126	M: 34 (15-45)<br>P: 34 (20-40)	8	65	71,85	DSM-IV single or recurrent major depressive episode	DB-RCT	Organon Pharmac euticals
Schatzberg 2006 (38)	Venlafaxine: n= 104<br>Placebo: n= 96<br>Fluoxetine: n= 100	V: 225 (75-225)<br>F: - (20-60)	8	65	71	DSM-IV unipolar depression (single or recurrent, nonpsychotic)	DB-RCT	Wyeth Re-search
Schifano 1990 (39)	Mianserin: n= 25<br>Maprotiline: n= 23	Mi: 67,6-90<br>Ma: 112,5-150	4	65	75,41	DSM-III a major depressive episode	DB-RCT	No sponsor stated
Schoene 1993 (40)	Fluoxetine: n= 52<br>Paroxetine: n= 54	F: 20-60<br>P: 20-40	6	61	74,01	DSM-III-R current episode of major depression	DB-RCT	No sponsor stated
Schweizer 1998 (41)	Imipramine: n=60<br>Buspirone: n= 57<br>Placebo: n= 60	I: 89 (25-150)<br>B: 38 (10-60)	8	65	72	DSM-III-R unipolar major depression	DB-RCT	Bristol-Myers Squibb
Smeraldi 1997 (42)	Venlafaxine: n= 55<br>Clomipramine: n= 58<br>Trazodone: n= 57	V: 75-150<br>C: 50-100<br>T: 150-300	6	65	71	DSM-III-R major depression	DB-RCT	No sponsor stated
Study 032a (43)	Reboxetine: n= 24<br>Placebo: n= 26	R: 4-6	8	63	79,96	DSM-III-R Major Depressive Disorder not accompanied by	DB-RCT	No sponsor stated

						psychotic features		
Tignol 1998 (44)	Imipramine: n= 107<br>Milnacipran: n= 112	I: 75-100<br>M: 75-100	8	65	74,10	DSM-III-R MDE with or without melancholia and without psychotic features	DB-RCT	No sponsor stated

DB-RCT: Double-Blind Randomized Trial, SB-RCT: Single-Blind Randomized Trial, DSM: Diagnostic and Statistical Manual of Mental Disorders, ICD: International Statistical Classification of Diseases and Related Health Problems

References

1. Allard P, Gram L, Timdahl K, Behnke K, Hanson M, Søgaard J. Efficacy and tolerability of venlafaxine in geriatric outpatients with major depression: a double-blind, randomised 6-month comparative trial with citalopram. International journal of geriatric psychiatry 2004; 19(12):1123–30.

2. Anon. Venlafaxine and nortriptyline similar in depression. Brown University Geriatric Psychopharmacology Update 2003; 7(3):7.

3. Bocksberger JP, Gachoud JP, Richard J, Dick P. Comparison of the efficacy of moclobemide and fluvoxamine in elderly patients with a severe depressive episode. European psychiatry 1993; 8(6):319–24.

4. Brion S, Audrain S, Bodinat C de. Major depressive episodes in patients over 70 years of age. Evaluation of the efficiency and acceptability of tianeptine and mianserin. Presse medicale (Paris, France : 1983) 1996; 25(9):461–8.

5. Cassano GB, Puca F, Scapicchio PL, Trabucchi M, Italian Study Group on Depression in Elderly, Patients. Paroxetine and fluoxetine effects on mood and cognitive functions in depressed nondemented elderly patients. Journal of clinical psychiatry 2002; 63(5):396–402.

6. Chen YM, Huang XM, Thompson R, Zhao YB. Clinical features and efficacy of escitalopram treatment for geriatric depression. Journal of international medical research 2011; 39(5):1946–53.

7. Cohn CK, Shrivastava R, Mendels J, Cohn JB, Fabre LF, Claghorn JL et al. Double-blind, multicenter comparison of sertraline and amitriptyline in elderly depressed patients. Journal of clinical psychiatry 1990; 51 Suppl B(Suppl B):28–33.

8. Dorman T. Sleep and paroxetine: a comparison with mianserin in elderly depressed patients. Int Clin Psychopharmacol 1992; 6 Suppl 4(Suppl 4):53–8.

9. EUCTR-001829-33-FR. Duloxetine Versus Placebo in the Long-Term Treatment of Patients with Late-Life Major Depression - HMFA: https://www.clinicaltrialsregister.eu/ctr-search/search?query=eudract_number:2008-001829-33; 2008.

10. EUCTR-003821-25-DK. A 12 week multi-centre, randomized, double-blind, placebo controlled evaluation of the most efficacious and tolerable dose of escitalopram in the treatment of elderly patients. - Double-blind placebo controlled study of escitalopram in the treatment of depression in

the elderly: https://www.clinicaltrialsregister.eu/ctr-search/search?query=eudract_number:2005-003821-25; 2005.

11. EUCTR-005612-26-SK. Efficacy and safety of tianeptine oral administration (25 to 50 mg/day) in elderly patients suffering from Major Depressive Disorder. A 8-week, randomized, double-blind, flexible-dose, parallel groups, placebo-controlled, international, multicentre study with escitalopram as active control, followed by an optional double-blind extension treatment period of 16 weeks: https://www.clinicaltrialsregister.eu/ctr-search/search?query=eudract_number:2012-005612-26; 2013.

12. Finkel SI, Richter EM, Clary CM. Comparative efficacy and safety of sertraline versus nortriptyline in major depression in patients 70 and older. International psychogeriatrics / IPA 1999; 11(1):85–99.

13. Finkel SI, Richter EM, Clary CM, Batzar E. Comparative efficacy of sertraline vs. fluoxetine in patients age 70 or over with major depression. American journal of geriatric psychiatry 1999; 7(3):221–7.

14. Geretsegger C, Stuppaeck CH, Mair M, Platz T, Fartacek R, Heim M. Multicentre double-blind study of paroxetine and amitriptyline in elderly depressed inpatients. Psychopharmacology 1995; 119(3):277–81.

15. GlaxoSmithKline. A Double Blind Comparative Study of the Effects of Paroxetine and Clomipramine on Cognitive Function in Elderly Patients with Major Depression. GSK - Clinical Study Register [www.gsk-clinicalstudyregister.com] 1991.

16. GlaxoSmithKline. A double-blind, beetween patient, multicentre study in general practice comparing the efficacy and tolerability of paroxetine with those of dothiepin in the treatment of elderly depressed patients. GSK - Clinical Study Register [www.gsk-clinicalstudyregister.com] 1991.

17. GlaxoSmithKline. A Double-Blind, Multicentre Study Comparing the Efficacy, Tolerability and Effects on Cognitive Function of Paroxetine With Those of Lofepramine in Elderly Depressed Hospital In- or Out-Patients. GSK - Clinical Study Register [www.gsk-clinicalstudyregister.com] 1993.

18. Guelfi JD, Bouhassira M, Bonett-Perrin E, Lancrenon S. Study of the efficacy of fluoxetine versus tianeptine in the treatment of elderly depressed patients, followed in general practice. Encephale 1999; 25(3):265–70.

19. Heun R, Ahokas A, Boyer P, Gimenez-Montesinos N, Pontes-Soares F, Olivier V. The efficacy of agomelatine in elderly patients with recurrent major depressive disorder: A placebo-controlled study [ISRCTN57507360]. Journal of clinical psychiatry 2013; 74(6):587–94.

20. Hewett K, Chrzanowski W, Jokinen R, Felgentreff R, Shrivastava RK, Gee MD et al. Double-blind, placebo-controlled evaluation of extended-release bupropion in elderly patients with major depressive disorder. Journal of psychopharmacology (Oxford, England) 2010; 24(4):521–9. Available from: URL: https://clinicaltrials.gov/ct2/show/NCT00093288.

21. Hutchinson DR, Tong S, Moon CA, Vince M, Clarke A. Paroxetine in the treatment of elderly depressed patients in general practice: a double-blind comparison with amitriptyline. International clinical psychopharmacolology 1992; 6 Suppl 4(Suppl 4):43–51.

22. Jansen RW, Mehagnoul-Schipper D, Hulsbos P, Jellesma-Eggenkamp M, Hoefnagels W. Effect of paroxetine and nortriptyline on orthostatic and postprandial hypotension in patients 70 years of age with depressive disorders [conference presentation]. International Psychogeriatrics [abstracts from

The International Psychogeriatric Association Eleventh International Congress Chicago, United States 17-22 August 2003] 2003; 15(Suppl 2).

23. Karlsson I, Godderis J, Augusto De Mendonca Lima, C., Nygaard H, Simanyi M, Taal M et al. A randomised, double-blind comparison of the efficacy and safety of citalopram compared to mianserin in elderly, depressed patients with or without mild to moderate dementia. International journal of geriatric psychiatry 2000; 15(4):295–305.

24. Kasper S, Swart H de, Friis Andersen H. Escitalopram in the treatment of depressed elderly patients. American journal of geriatric psychiatry 2005; 13(10):884–91.

25. Katona C, Bercoff E, Chiu E, Tack P, Versiani M, Woelk H. Reboxetine versus imipramine in the treatment of elderly patients with depressive disorders: a double-blind randomised trial. J Affect Disord 1999; 55(2-3):203–13.

26. Katona C, Hansen T, Olsen CK. A randomised, double-blind, placebocontrolled, active-referenced study of the multimodal antidepressant Lu AA21004 in the treatment of elderly depressed patients. European neuropsychopharmacology 2012; 22:S258-S259.

27. Kyle CJ, Petersen H, Hopfner E. Citalopram treats major depression in the elderly without causing anticholinergic side-effects. XXIst Collegium Internationale Neuro Psychopharmacologicum, Glasgow, Scotland. 12th 16th July, 1998. 1998.

28. Mahapatra SN, Hackett D. A randomised, double-blind, parallel-group comparison of venlafaxine and dothiepin in geriatric patients with major depression. International journal of clinical practice 1997; 51(4):209–13.

29. Nair NP, Amin M, Schwartz G, Dastoor D, Thavundayil JX, Mirmiran J et al. A comparison of the cardiac safety and therapeutic efficacy of trimipramine versus doxepin in geriatric depressed patients. Journal of the American Geriatrics Society 1993; 41(8):863–7.

30. NCT00130455. Treatment of Depression in the Elderly: https://ClinicalTrials.gov/show/NCT00130455; April April. Available from: URL: https://ClinicalTrials.gov/show/NCT00130455.

31. Newhouse P, Finkel S, Richter E. SSRIs in the treatment of depressed out patients aged 70 years and older. 8th ECNP (European College of Neuropsychopharmacology) Congress, Venice, Italy 1995.

32. Phanjoo AL, Wonnacott S, Hodgson A. Double-blind comparative multicentre study of fluvoxamine and mianserin in the treatment of major depressive episode in elderly people. Acta psychiatrica Scandinavica 1991; 83(6):476–9.

33. Rahman MK, Akhtar MJ, Savla NC, Sharma RR, Kellett JM, Ashford JJ. A double-blind, randomised comparison of fluvoxamine with dothiepin in the treatment of depression in elderly patients. British journal of clinical practice 1991; 45(4):255–8.

34. Raskin J, Wiltse CG, Siegal A, Sheikh J, Xu J, Dinkel JJ et al. Efficacy of duloxetine on cognition, depression, and pain in elderly patients with major depressive disorder: an 8-week, double-blind, placebo-controlled trial. American journal of psychiatry 2007; 164(6):900–9.

35. Robinson M, Oakes TM, Raskin J, Liu P, Shoemaker S, Nelson JC. Acute and long-term treatment of late-life major depressive disorder: duloxetine versus placebo. American journal of geriatric psychiatry 2014; 22(1):34–45.

36. Roose SP, Sackeim HA, Krishnan KR, Pollock BG, Alexopoulos G, Lavretsky H et al. Antidepressant pharmacotherapy in the treatment of depression in the very old: a randomized, placebo-controlled trial. American journal of psychiatry 2004; 161(11):2050–9.

37. Schatzberg AF, Kremer C, Rodrigues HE, Murphy GM, Mirtazapine vs. Paroxetine Study, Group. Double-blind, randomized comparison of mirtazapine and paroxetine in elderly depressed patients. American journal of geriatric psychiatry 2002; 10(5):541–50.

38. Schatzberg A, Roose S. A double-blind, placebo-controlled study of venlafaxine and fluoxetine in geriatric outpatients with major depression. American journal of geriatric psychiatry 2006; 14(4):361–70.

39. Schifano F, Garbin A, Renesto V, Dominicis MG de, Trinciarelli G, Silvestri A et al. A double-blind comparison of mianserin and maprotiline in depressed medically ill elderly people. Acta psychiatrica Scandinavica 1990; 81(3):289–94.

40. Schone W, Ludwig M. A double-blind study of paroxetine compared with fluoxetine in geriatric patients with major depression. Journal of clinical psychopharmacology 1993; 13(6 Suppl 2):34S-39S.

41. Schweizer E, Rickels K, Hassman H, Garcia-Espana F. Buspirone and imipramine for the treatment of major depression in the elderly. Journal of clinical psychiatry 1998; 59(4):175–83.

42. Smeraldi E, Aguglia A, Cattaneo M, Cerati C, Covelli C, Del Zompo M et al. Double-blind, randomized study of venlafaxine, clomipramine, and trazodone in geriatric patients with major depression. 10th European College of Neuropsychopharmacology Congress. Vienna, Austria. 13th 17th September 1997. 1997.

43. Institut für Qualität und Wirtschaftlichkeit im Gesundheitswesen. study032a 1995.

44. Tignol J, Pujol-Domenech J, Chartres JP, Leger JM, Pletan Y, Tonelli I et al. Double-blind study of the efficacy and safety of milnacipran and imipramine in elderly patients with major depressive episode. Acta psychiatrica Scandinavica 1998; 97(2):157–65.
