# Supplementary material for: How well do elderly patients with major depressive disorder respond to antidepressants: a systematic review and single-group meta-analysis
Source: BMC Psychiatry. 2020 Mar 4;20:102. doi: 10.1186/s12888-020-02514-2 (PMC7057600; doi:10.1186/s12888-020-02514-2)

Risk of bias assessment

Risk of bias summary

	Random sequence generation (selection bias)	Allocation concealment (selection bias)	Blinding of participants and personnel (performance bias)	Blinding of outcome assessment (detection bias)	Incomplete outcome data (attrition bias)	Selective reporting (reporting bias)	Other bias
Allard2004	?	?	+	+	+	+	+
Anon 2003	?	?	−	−	+	+	+
Bocksberger 1993	?	?	+	+	+	+	+
Brion 1996	?	?	?	?	+	+	+
Cassano2002	?	?	?	?	+	+	+
Chen 2011	+	?	+	+	−	+	+
Cohn1990	?	?	?	?	+	+	+
Dorman 1992	?	?	+	+	+	+	+
EUCTR-001829-33-FR 2008	?	?	?	?	+	+	+
EUCTR-003821-25-DK 2005	?	?	?	?	?	?	?
EUCTR-005612-26-SK 2013	?	?	+	+	+	+	+
Finkel 1999a	+	+	+	+	+	−	+
Finkel 1999b	?	+	+	+	+	+	+
Geretsegger1995 (MY-060/BRL-029060/1)	?	?	+	+	+	−	+
GlaxoSmithKline 1991	?	?	?	+	+	+	+
GlaxoSmithKline 1991b	?	?	?	?	+	+	?
GlaxoSmithKline 1993	?	?	?	?	+	?	?
Guelfi 1999	?	?	?	?	+	+	+
Heun2013 (ISRCTN57507360)	+	+	?	?	+	+	+
Hewett2010	?	?	+	+	+	+	+
Hutchinson1992	?	?	+	+	+	+	+
Jansen 2003	?	?	+	+	?	?	?
Karlsson 2000	?	?	+	+	+	+	+
Kasper2005	?	?	?	?	+	+	+
Katona 1999	?	?	?	?	+	+	+
Katona2012 (12541A, NCT00811252)	+	+	+	+	+	+	+
Kyle1998 (Study 92032 - FDA)	?	?	+	+	+	+	+
Mahapatra 1997	?	?	?	?	+	+	+
Nair 1993	?	?	+	+	−	−	+
NCT00130455 2006	?	?	?	?	?	?	?
Newhouse 1995	?	?	+	+	+	+	+
Phanjoo 1991	?	?	?	?	+	+	+
Rahman 1991	?	?	+	+	+	+	+
Raskin2007 (HMBV) (NCT00062673)	?	?	?	?	+	+	+
Robinson2014 (NCT00406848)	+	+	+	+	+	+	+
Roose2004 (CIT-MD-03)	+	+	?	?	+	+	+
Schatzberg2002 (003-901)	?	?	+	+	+	+	+
Schatzberg2006a	+	+	+	+	+	+	+
Schifano 1990	+	+	+	+	+	+	+
Schoene1993 (Geretsegger1994 MY1021/BRC)	?	?	?	?	+	+	?
Schweizer 1998	?	?	?	?	+	+	+
Smeraldi1997	?	?	?	?	?	?	+
Study 032a (CTN032-FCE20124)	+	+	−	−	+	+	+
Tignol 1998	?	?	?	?	+	−	+

Risk of bias graph

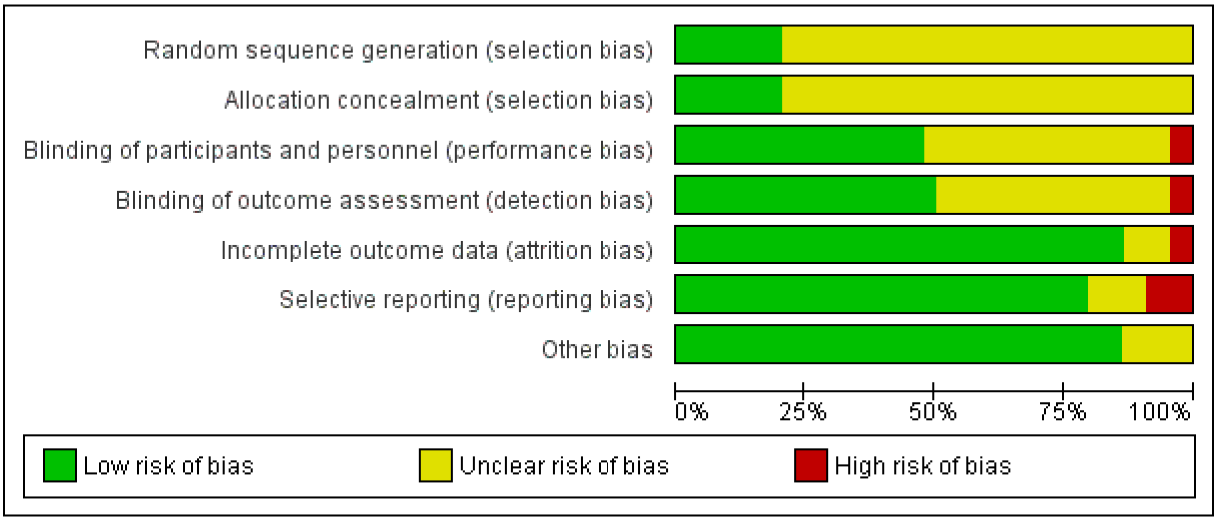

Supplement: Supplementary file 2 — Additional file 2. Risk of bias assessment (pdf). [file 12888_2020_2514_MOESM2_ESM.pdf]
