# Supplementary material for: How well do elderly patients with major depressive disorder respond to antidepressants: a systematic review and single-group meta-analysis
Source: BMC Psychiatry. 2020 Mar 4;20:102. doi: 10.1186/s12888-020-02514-2 (PMC7057600; doi:10.1186/s12888-020-02514-2)
Supplement: Supplementary file 3 — Additional file 3. Sensitivity analysis (pdf). [file 12888_2020_2514_MOESM3_ESM.pdf]

Sensitivity analysis excluding imputed response rates

Study name	drug	Event rate	Lower limit	Upper limit	Z-Value	p-Value
Allard2004	Citalopram	0.667	0.553	0.764	2.830	0.005
Allard2004	Venlafaxine	0.645	0.532	0.744	2.487	0.013
Brion 1996	Mianserin	0.557	0.461	0.648	1.163	0.245
Brion 1996	Tianeptine	0.608	0.540	0.672	3.088	0.002
Cassano2002	Paroxetine	0.285	0.212	0.370	-4.614	0.000
Cassano2002	Fluoxetine	0.126	0.077	0.199	-7.011	0.000
Chen 2011	Escitalopram	0.586	0.404	0.748	0.924	0.356
Cohn1990	Sertraline	0.522	0.445	0.598	0.552	0.581
Cohn1990	Amitriptyline	0.500	0.392	0.608	0.000	1.000
Dorman 1992	Paroxetine	0.483	0.311	0.659	-0.186	0.853
Dorman 1992	Mianserin	0.179	0.076	0.364	-3.093	0.002
Finkel 1999a	Fluoxetine	0.424	0.270	0.595	-0.867	0.386
Finkel 1999a	Sertraline	0.571	0.420	0.711	0.923	0.356
Finkel 1999b	Sertraline	0.487	0.336	0.640	-0.160	0.873
Finkel 1999b	Nortriptyline	0.243	0.132	0.405	-2.962	0.003
Geretsegger1995	Paroxetine	0.409	0.275	0.558	-1.199	0.230
Geretsegger1995	Amitriptyline	0.383	0.256	0.528	-1.589	0.112
GlaxoSmithKline 1991	Dothiepin	0.522	0.404	0.639	0.366	0.714
GlaxoSmithKline 1991	Paroxetine	0.433	0.320	0.553	-1.096	0.273
GlaxoSmithKline 1993	Lofepramine	0.531	0.392	0.665	0.428	0.668
GlaxoSmithKline 1993	Paroxetine	0.526	0.398	0.651	0.397	0.691
Guelfi 1999	Fluoxetine	0.705	0.618	0.779	4.387	0.000
Guelfi 1999	Tianeptine	0.565	0.473	0.653	1.395	0.163
Heun2013	Agomelatine	0.583	0.503	0.659	2.025	0.043
Hewett2010	Bupropion	0.514	0.447	0.581	0.412	0.680
Hutchinson1992	Amitriptyline	0.844	0.675	0.933	3.464	0.001
Hutchinson1992	Paroxetine	0.759	0.633	0.852	3.732	0.000
Karlsson 2000	Mianserin	0.630	0.556	0.699	3.381	0.001
Karlsson 2000	Citalopram	0.571	0.493	0.644	1.795	0.073
Kasper2005	Fluoxetine	0.372	0.301	0.448	-3.242	0.001
Kasper2005	Escitalopram	0.448	0.376	0.523	-1.362	0.173
Katona 1999	Reboxetine	0.532	0.438	0.624	0.670	0.503
Katona 1999	Imipramine	0.523	0.429	0.615	0.479	0.632
Katona2012	Duloxetine	0.616	0.536	0.690	2.822	0.005
Katona2012	Vortioxetine	0.526	0.447	0.603	0.640	0.522
Kyle1998	Amitriptyline	0.667	0.596	0.731	4.456	0.000
Kyle1998	Citalopram	0.682	0.610	0.746	4.743	0.000
Mahapatra 1997	Dothiepin	0.583	0.441	0.713	1.149	0.250
Mahapatra 1997	Venlafaxine	0.591	0.442	0.725	1.199	0.230
Nair 1993	Doxepin	0.526	0.311	0.732	0.229	0.819
Nair 1993	Trimipramine	0.667	0.429	0.842	1.386	0.166
Newhouse 1995	Fluoxetine	0.606	0.434	0.756	1.209	0.227
Newhouse 1995	Sertraline	0.810	0.663	0.902	3.682	0.000
Raskin2007	Duloxetine	0.362	0.300	0.430	-3.910	0.000
Roose2004	Citalopram	0.405	0.305	0.513	-1.735	0.083
Schatzberg2002	Mirtazapine	0.563	0.476	0.646	1.410	0.158
Schatzberg2002	Paroxetine	0.476	0.391	0.563	-0.534	0.593
Schatzberg2006a	Venlafaxine	0.394	0.305	0.491	-2.141	0.032
Schatzberg2006a	Fluoxetine	0.370	0.281	0.468	-2.570	0.010
Schifano 1990	Maprotiline	0.304	0.153	0.515	-1.824	0.068
Schifano 1990	Mianserin	0.480	0.296	0.669	-0.200	0.842
Schoene1993	Paroxetine	0.352	0.237	0.487	-2.144	0.032
Schoene1993	Fluoxetine	0.154	0.079	0.279	-4.435	0.000
Schweizer 1998	Imipramine	0.600	0.472	0.715	1.539	0.124
Smeraldi1997	Trazodone	0.579	0.448	0.699	1.187	0.235
Smeraldi1997	Clomipramine	0.690	0.560	0.795	2.813	0.005
Smeraldi1997	Venlafaxine	0.745	0.615	0.843	3.471	0.001
Study 032a	Reboxetine	0.167	0.064	0.369	-2.938	0.003
Tignol 1997	Milnacipran	0.598	0.505	0.685	2.065	0.039
Tignol 1998	Imipramine	0.598	0.503	0.687	2.017	0.044
		0.517	0.481	0.552	0.912	0.362

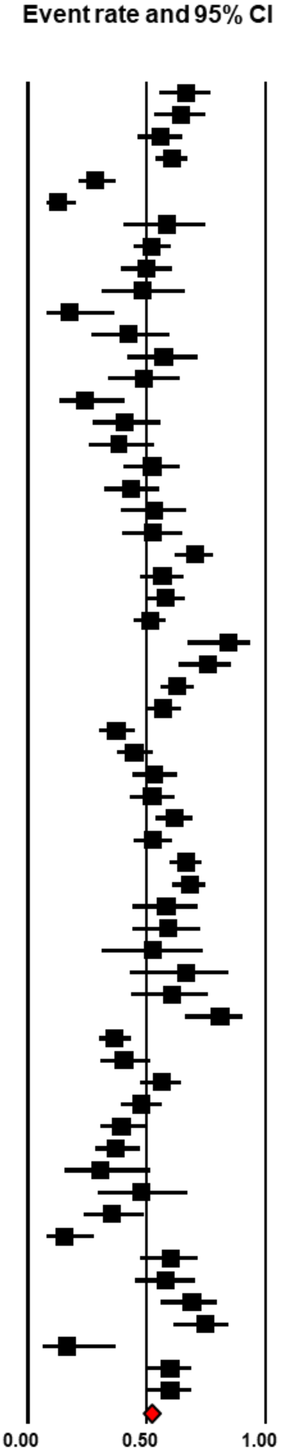

The squares represent the response rates and the horizontal lines reflect the 95% confidence interval. The red diamond corresponds to the overall response rate in the sensitivity analysis.

	Coefficient	*Lower limit*	*Upper limit*	*Z-value*	*P-value*
Exclusion of imputed response rates	0.52	0.48	0.55	0.912	0.362
