# Supplementary material for: How well do elderly patients with major depressive disorder respond to antidepressants: a systematic review and single-group meta-analysis
Source: BMC Psychiatry. 2020 Mar 4;20:102. doi: 10.1186/s12888-020-02514-2 (PMC7057600; doi:10.1186/s12888-020-02514-2)
Supplement: Supplementary file 4 — Additional file 4 Antidepressant subgroup p-values(pdf). [file 12888_2020_2514_MOESM4_ESM.pdf]

Antidepressant subgroup, p-values for the comparisons

	SSRI	TCA	SSNRI	SNRI	α2-Antagonist	MAO-Inhibitor
SSRI		0.061	0.895	0.502	0.671	**0.027**
TCA	0.061		0.277	0.289	0.317	0.116
SSNRI	0.895	0.277		0.488	0.840	**0.047**
SNRI	0.502	0.289	0.488		0.438	0.080
α2-Antagonist	0.671	0.317	0.840	0.438		0.053
MAO-Inhibitor	**0.027**	0.116	**0.047**	0.080	0.053	

MAO-inhibitors = Monoamine oxidase inhibitors, SNRI = Serotonin–norepinephrine reuptake inhibitor, SSNRI = Selective serotonin–norepinephrine reuptake inhibitor, SSRI = Selective serotonin reuptake inhibitor, TCA = Tricyclic antidepressant,
