# Supplementary material for: How well do elderly patients with major depressive disorder respond to antidepressants: a systematic review and single-group meta-analysis
Source: BMC Psychiatry. 2020 Mar 4;20:102. doi: 10.1186/s12888-020-02514-2 (PMC7057600; doi:10.1186/s12888-020-02514-2)
Supplement: Supplementary file 5 — Additional file 5. Small-study effect (pdf). [file 12888_2020_2514_MOESM5_ESM.pdf]

Small-study effect

1. Funnel plot

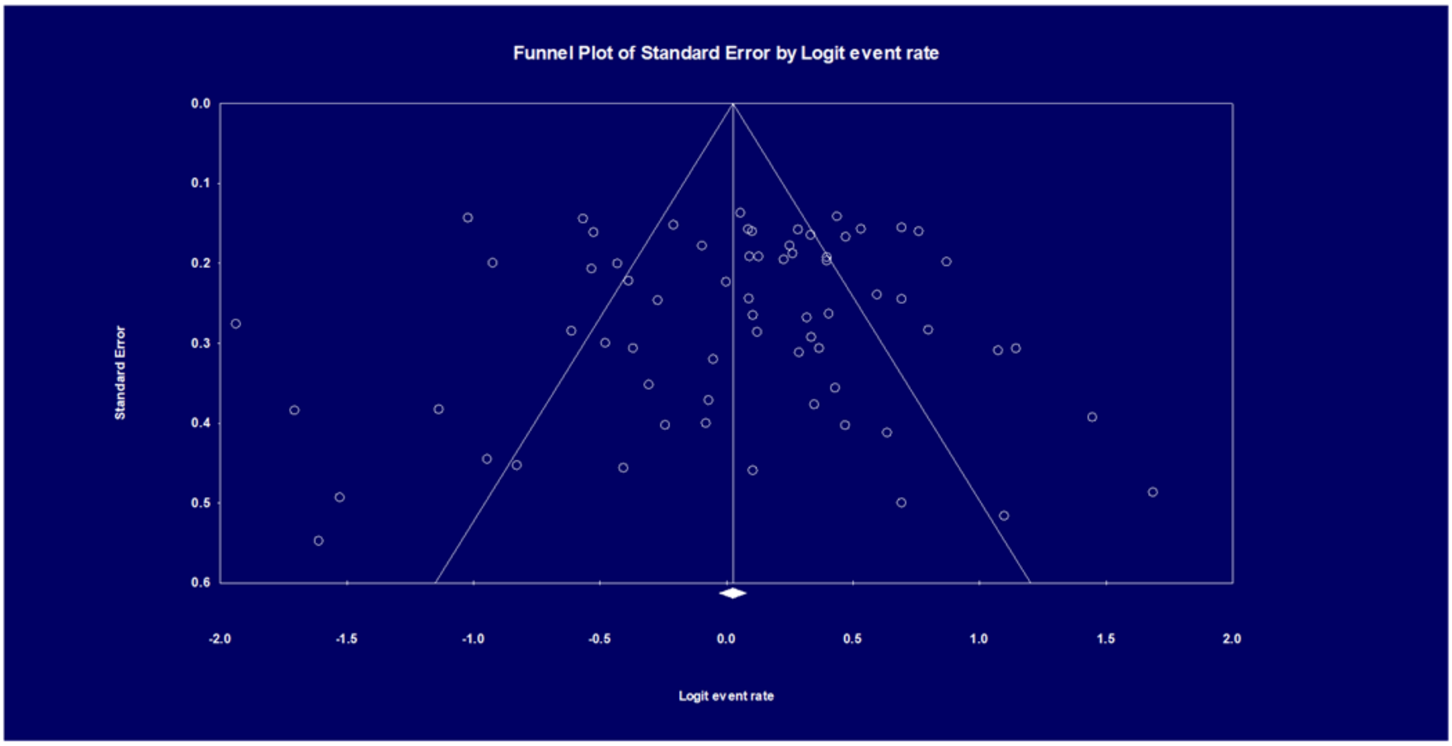

2. Egger´s regression intercept

Egger's regression intercept

Intercept	0,11173
Standard error	0,91987
95% lower limit (2-tailed)	-1,72486
95% upper limit (2-tailed)	1,94831
t-value	0,12146
df	66,00000
P-value (1-tailed)	0,45185
P-value (2-tailed)	0,90370
