# Supplementary material for: How well do elderly patients with major depressive disorder respond to antidepressants: a systematic review and single-group meta-analysis
Source: BMC Psychiatry. 2020 Mar 4;20:102. doi: 10.1186/s12888-020-02514-2 (PMC7057600; doi:10.1186/s12888-020-02514-2)
Supplement: Supplementary file 6 — Additional file 6. Response rates in adults with MDD (pdf). [file 12888_2020_2514_MOESM6_ESM.pdf]

Results of pooled response rate in general population with MDD

	Coefficient	*Lower limit*	*Upper limit*	*Z-value*	*P-value*
Response rate in general population with MDD	0.53	0.52	0.54	7.00	0.00

The calculated response rate is based on the published dataset by Cipriani and colleagues (1).

References

1. Cipriani A, Furukawa TA, Salanti G, Chaimani A, Atkinson LZ, Ogawa Y et al. Comparative efficacy and acceptability of 21 antidepressant drugs for the acute treatment of adults with major depressive disorder: A systematic review and network meta-analysis. The Lancet 2018; 391(10128):1357–66.
