# Supplementary material for: How well do elderly patients with major depressive disorder respond to antidepressants: a systematic review and single-group meta-analysis
Source: BMC Psychiatry. 2020 Mar 4;20:102. doi: 10.1186/s12888-020-02514-2 (PMC7057600; doi:10.1186/s12888-020-02514-2)
Supplement: Supplementary file 7 — Additional file 7. Placebo response rates (pdf). [file 12888_2020_2514_MOESM7_ESM.pdf]

## Response Rates for patients in the placebo arm

### 1. Forest plot

| Study name | Statistics for each study | | | | | Event rate and 95% CI |
|---|---|---|---|---|---|---|
| | Event rate | Lower limit | Upper limit | Z-Value | p-Value | |
| Chen 2011 | 0,019 | 0,001 | 0,236 | -2,781 | 0,005 | |
| EUCTR-001829-33-FR 2008 | 0,215 | 0,151 | 0,297 | -5,854 | 0,000 | |
| Heun2013 | 0,380 | 0,275 | 0,498 | -1,998 | 0,046 | |
| Hewett2010 | 0,418 | 0,353 | 0,486 | -2,347 | 0,019 | |
| Kasper2005 | 0,472 | 0,400 | 0,545 | -0,745 | 0,456 | |
| Katila 2013 | 0,302 | 0,238 | 0,375 | -5,037 | 0,000 | |
| Katona2012 | 0,352 | 0,278 | 0,433 | -3,516 | 0,000 | |
| Raskin2007 | 0,183 | 0,120 | 0,269 | -5,904 | 0,000 | |
| Robinson2014 | 0,215 | 0,151 | 0,297 | -5,854 | 0,000 | |
| Roose2004 | 0,374 | 0,281 | 0,477 | -2,384 | 0,017 | |
| Schatzberg2006a | 0,417 | 0,323 | 0,517 | -1,625 | 0,104 | |
| Schweizer 1998 | 0,350 | 0,241 | 0,478 | -2,287 | 0,022 | |
| Study 032a | 0,192 | 0,082 | 0,387 | -2,884 | 0,004 | |
| **overall** | **0,317** | **0,263** | **0,378** | **-5,607** | **0,000** | |

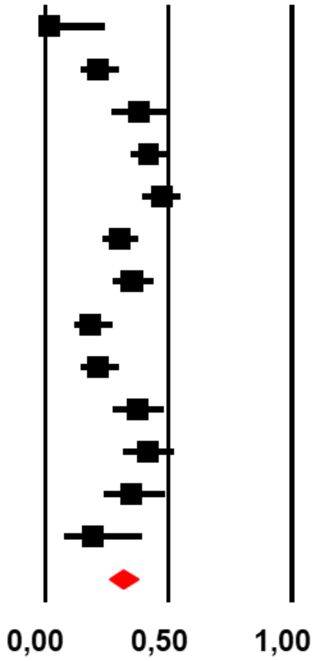


The squares represent the response rates and the horizontal lines reflect the 95% confidence interval. The red diamond corresponds to the overall response rate. CI = Confidence interval

### 2. Table

| | *Coefficient* | *Lower limit* | *Upper limit* |
|---|---|---|---|
| Placebo (N=13) | 0.32 | 0.26 | 0.38 |

N: Number of study arms
